# Review of Recent Improvements in Carbon Ion Radiation Therapy in the Treatment of Glioblastoma

**DOI:** 10.1016/j.adro.2024.101465

**Published:** 2024-02-08

**Authors:** Fereshteh Koosha, Mahdieh Ahmadikamalabadi, Mohadesseh Mohammadi

**Affiliations:** a Department of Radiology Technology, School of Allied Medical Sciences, Shahid Beheshti University of Medical Sciences, Tehran, Iran; b Social Determinants of Health Research Center, Rafsanjan University of Medical Sciences, Rafsanjan, Iran; c Radiology Department, School of Paramedical Sciences, Rafsanjan University of Medical Sciences, Rafsanjan, Iran

## Abstract

**Purpose:**

This article provides an overview of the physical and biologic properties of carbon ions, followed by an examination of the latest clinical outcomes in patients with glioma who have received carbon ion radiation therapy.

**Methods and Materials:**

According to thee articles that have been reviewed, glioma represents the predominant form of neoplastic growth in the brain, accounting for approximately 51% of all malignancies affecting the nervous system. Currently, high-grade glioma, specifically glioblastoma, comprises 15% of cases and is associated with a high mortality rate. The development of novel drugs for the treatment of high-grade tumors has been impeded by various factors, such as the blood-brain barrier and tumor heterogeneity, despite numerous endeavors. According to the definition of tumor grade established by the World Health Organization, the conventional treatment involves surgical resection followed by adjuvant radiation and chemotherapy. Despite numerous attempts in photon radiation therapy to apply the highest possible dose to the tumor site while minimizing damage to healthy tissue, there has been no success in increasing patient survival. The primary cause of resistance to conventional radiation therapy methods, namely x-ray and gamma-ray, is attributed to the survival of radio-resistant glioma stem cells, which have the potential to trigger a recurrence of tumors. Particle beams, such as protons and carbon ions, can deposit the highest dose to a confined region, thus offering a more accurate dose distribution compared with photon beams.

**Results:**

Carbon ions exhibit higher linear energy transfer and relative biologic effectiveness compared with photons, potentially enabling them to overcome radio-resistant tumor cells.

**Conclusions:**

Therefore, it can be hypothesized that carbon ion radiation therapy may show superior efficacy in destroying neoplastic cells with reduced negative outcomes compared with x-ray radiation therapy.

## Introduction

The primary approach for managing glioblastoma, the most prevalent and severe primary brain tumor in adults, is radiation therapy. There are several different types of gliomas, from the relatively indolent pilocytic astrocytoma (World Health Organization [WHO] grade 1) to the nearly always deadly glioblastoma (WHO grade IV).^1^ The therapeutic regimen involves a series of interventions, beginning with surgical debulking, followed by ionizing radiation (IR) and alkylating chemotherapy with temozolomide. The goal of this approach is to eliminate any remaining infiltrative tumor cells that may be invading surrounding normal tissue. Despite a multifaceted approach, the median survival time for newly diagnosed glioblastoma multiform patients is nearly 14 months.^2^^,^^3^ One of the most efficient ways to treat both primary and metastatic solid tumors, as well as microscopic tumor expansions, is radiation therapy (RT). Despite advancements in the technical aspects of delivering doses to tumors, it remains challenging to prevent radiation exposure to critical organs and tissues surrounding the tumor. This can lead to various short- and long-term complications and sequelae. The adverse effects of radiation have a significant impact on the patient's quality of life. To prevent the radiation dose of normal organs and tissues surrounding the tumor from surpassing the tolerance level, it is necessary to decrease the radiation dose administered to the target area. This decrease in radiation dose can lead to a reduction in the rate of local control, as reported in previous studies.^4^

Particle radiation therapy, including the use of proton beams and heavy ions (primarily carbon ions), has been implemented in clinical practice in various countries, such as the United States, Japan, and Germany, during the past few decades. According to data published by the Particle Therapy Cooperative Group, the global number of patients treated with particle therapy exceeded 360,000 by the end of 2022. This treatment includes approximately 312,000 individuals treated with protons and 46,800 with carbon ions. Because of its high relative biologic effect (RBE), high linear energy transfer (LET), enhanced dose conformity, and decreased total dose to normal tissue, carbon ion radiation therapy (CIRT) has been proposed as a viable technique for cancer treatment.^5^ Carbon ions exhibit unique physical properties due to their inverted dose profile, whereby low radiation doses are localized within the entry channel of the beam, and high doses are deposited at greater depths.

The Bragg peak phenomenon can be used to accurately target radiation to a specific lesion by modulating the energy of the particle beam, taking advantage of the sharp dose reduction that follows the peak. Consequently, CIRT can accurately focus on malignant tissues while preserving adjacent healthy tissues, facilitating the escalation of dosage and mitigating adverse reactions.^6^ The clinical investigation of carbon ion radiation therapy is underway for the treatment of various malignant tumors, such as glioblastoma.^7^^,^^8^ This research reviews the physical and biologic properties of carbon ions and focuses on recent clinical outcomes in patients with glioma who received carbon ion therapy.

### Physical properties of carbon ions

**Figure 1:**
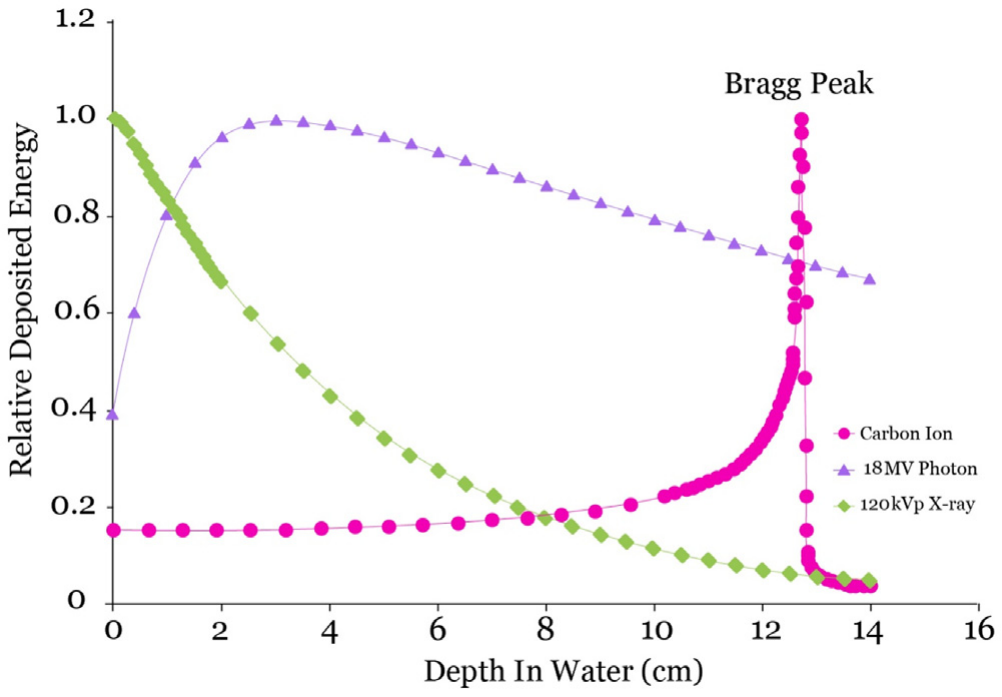
Comparison of percentage depth dose curves of carbon ion beams versus high (18 MV) and low (120 kVp) energy photon beams.^11^

Treatment with carbon ions results in a variety of distinct and radiobiologically useful physical properties (Table 1). Carbon ions demonstrate a distinctive energy distribution pattern in depth, commonly referred to as the "Bragg peak." This phenomenon results in the deposition of minimal energy levels in tissues that are near the intended target, and most of the energy is released precisely at the target site (Fig. 1). Upon penetrating matter, carbon ions promptly initiate the transfer of kinetic energy to the medium through which they traverse.^8^ How much energy moves from the ion to the medium depends on the rate of this energy loss. This energy transfer is called the LET, and it becomes larger as the particle's velocity decreases until all its kinetic energy is used up and the particle stops moving. The procedure generates a distinctive depth dose curve, wherein a minimal dose is administered in the shallower regions of the track but experiences a sudden escalation and culminates as the particle reaches its cessation point. The administered dosage subsequently experiences a significant decrease. The phenomenon of an abrupt and drastic deposition of energy within a specific range is commonly known as the “Bragg peak.” Although, unlike protons, energy is deposited distally due to nuclear fragmentation, distal tissues absorb minimal energy.^9^ Furthermore, it has been observed that heavy ions, specifically carbon, exhibit a more pronounced lateral dose penumbra at increased depths compared with photons or protons.^10^^,^^11^ In comparison to carbon ions, photons exhibit a relatively shallow depth dose maximum, typically limited to a maximum depth of approximately 3 to 4 cm. This presents a significant obstacle when attempting to treat tumors located at greater depths, as it becomes difficult to avoid damaging the surrounding healthy tissues both proximal and distal to the target area. The depth to which a charged particle can penetrate depends on how fast it travels at the outset. Synchrotrons and cyclotrons can be used to generate monoenergetic, narrow beams of carbon ions for medical applications. In clinical applications, the longitudinal and lateral spread of beams is used to generate a spread-out Bragg peak (SOBP), as depicted in Fig. 1. The high-dose regions are then contoured to conform to the target volume by appropriate shaping. The use of particle beams with variable initial kinetic energies, appropriately weighted relative to each other, can result in the creation of a uniform dose zone in the depth or direction of the beam, thereby covering the treated lesion regardless of the shape of the physical dose distribution. The generation of SOBP comprising charged particles presents numerous benefits in the field of particle therapy. The initial factor pertains to the increased proportion of administered dosage that is targeted toward the tumor in comparison to the surrounding healthy tissue near the tumor. Subsequently, a reduced amount of the administered dose is conveyed to healthy tissues located beyond the Bragg peak's posterior region, thereby facilitating further preservation of healthy tissues situated at the distal periphery of the neoplasm. However, because energy loss is stochastic when ions pass through tissue, not all ions halt at the same depth. This range uncertainty causes the Bragg peak to broaden in the longitudinal direction and decreases as the therapeutic ion's atomic mass increases. One notable differentiation between heavily charged particles and photons or protons is the reduced occurrence of multiple coulomb scattering during their traversal through a medium.

**Table 1:** Summary of different clinical trials with carbon ions in treatment of glioblastoma

Study	Disease	Patients	Time range	Radiation Modality	Total dose/fractions	Prior treatment	Concurrent treatment	Outcome
Mizoe et al,^44^ 2007	Glioblastoma and Anaplastic glioma	48	1994 - 2002	Photon + Carbon ion radiotherapy + chemotherapy	10 MV x-ray (50 Gy/25 fractions); Nimustine hydrochloride (100 mg/m^2^ in weeks 1, 4, or 5 of XRT); carbon (from 16.8-24.8 Gy/8 fractions)	-	-	The median survival time: 17 mo for glioblastoma and 35 mo for anaplastic glioma; main side effect: bone marrow suppression No grade 3 or higher acute reaction
Qiu et al,^39^ 2022	Glioblastoma	16	2017 - 2019	Carbon ion radiotherapy + Proton radiotherapy	Carbon ion boost [9,12, 15, and 18 Gy] in 3 fractions before proton (60 Gy RBE in 30 fractions)	-	Chemotherapy during proton therapy (Temozolomide 75 mg/m^2^, 7 d/wk)	Median follow-up: 17.9 mo-PFS and OS at 12 mo: 50.6% and 78.6% respectively; no severe acute or late toxicities were perceived in doses (9, 12, 15 Gy)
Combs et al,^53^ 2013	Glioblastoma	32	1996 - 2011	Retrospective comparison between carbon boost and photon (or photon with chemotherapy)	Carbon ion boost versus (photon ± TMZ)	-	-	Median overall survival: 9 mo (photon); 14 mo (photon + chemotherapy) and 18 mo (carbon ions) Figure median PFS: 5 mo (photon); 6 mo (photon + chemotherapy) and 8 mo (carbon ions)
Kong et al,^54^ 2019	High-grade glioma	47	2015 - 2019	Proton alone and proton with carbon ion	PRT only (60 GyE/30 Fx/6 wk); PRT + CIRT (either PRT 50 Gy/25 Fx + CIRT 10-12 GyE/4-5 Fx, or PRT 60 GyE/30 Fx + CIRT 9-12 GyE/3 Fx)	-	Chemotherapy (Temozolomide)	1-y OS and PFS rates: 100% vs 75% (*P* = .049) and 100% vs 58% (*P* = .004) for grade 3 and 4 gliomas
Kong et al,^52^ 2020	Glioblastoma multiforme or anaplastic glioma	50	2015 - 2018	Proton radiotherapy (24 patients) and proton radiotherapy plus a carbon-ion radiotherapy (CIRT) boost (26 patients)	Proton radiotherapy (60 gray-equivalents in 30 daily fractions); proton radiotherapy plus a carbon-ion radiotherapy (CIRT) boost (various dose-escalating schemes)	-	Chemotherapy (Temozolomide) (first day 75 mg/m^2^, 7 d/wk, followed by at least 6 cycles of adjuvant treatment at 150-200 mg/m^2^ for 5 d during each 28-d cycle)	12-mo OS rate: 87.8% 18-mo OS rates: 72.8% 12-mo PFS rate: 74.2% 18-mo PFS rates: 59.8%
Combs et al,^36^ 2010	Glioblastoma	Accruing	Since 2010	Carbon ion boost / Proton boost	Carbon ion boost (18 Gy/6 fraction)/Proton boost (10 Gy /5 fractions)	Radiochem-otherapy with TMZ up to 50 Gy	Chemotherapy with TMZ (conventional dosing of 75 mg/m^2^ per d)	Awaiting results

*Abbreviations:* CIRT = carbon ion radiotherapy; Fx = fraction; OS = overall survival; PFS = progression free survival; PRT = proton radiotherapy; RBE = relative biologic effectiveness; TMZ = temozolomide; XRT = x-ray therapy.

LET refers to the amount of energy deposited by charged particles per unit length. This parameter is influenced by the charge and velocity of the particles. High LET radiation deposits dense energy along its path, and low LET radiation deposits dispersed energy. The tissue's entrance region exhibits lower LET values due to the high velocities of the particles. As the particle approaches deeper regions where the tumor is situated, its kinetic energy decreases, resulting in an increase in LET values. The clinical presentation of this phenomenon is that while proximal tissues may receive some dosage, particles passing through these tissues at shallower depths possess a lower LET and consequently inflict less harm than the high LET segment of the track that is deliberately situated within the tumor region. CIRT is a viable therapeutic option because it can deliver higher doses to targets while decreasing irradiation to organs at risk due to its higher LET compared with other radiation technologies and the properties of the Bragg peak. The incident ions are scattered laterally due to Coulomb scattering with the target's atomic nuclei. The depth of the ion stream is widened due to both Coulomb scattering and nuclear interactions, which produce secondary particles. The phenomenon has the potential to result in the disintegration of the cohesive forces between the nucleons, leading to the liberation of nuclear fragments originating from both the projectile and target nuclei. The charge number of projectile fragments is potentially variable, but it cannot exceed the charge of the primary. The production of secondary protons, helium, lithium, beryllium, and boron ions may occur in the presence of carbon ions. The fragments that are targeted are commonly considered to be of lesser significance, as their recoil energy is typically minimal, and their range is therefore limited, owing to the collision kinetics. In most cases, it is presumed that they are assimilated at the site of origin.^12^

### Radiobiological properties of carbon ions

**Figure 2:**
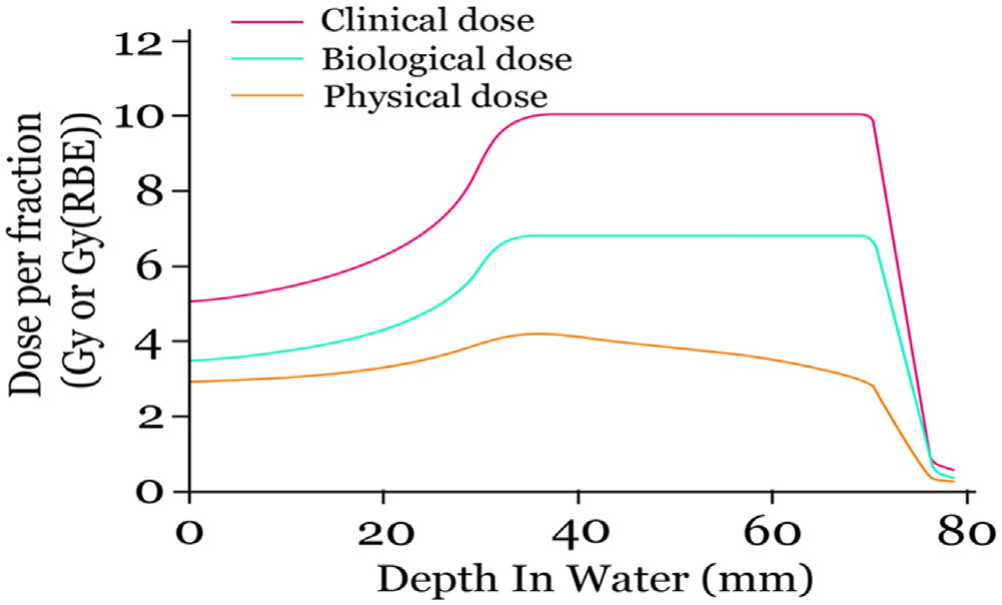
Physical, biologic, and clinical depth-dose distributions for carbon beam spread-out Bragg peak. The relative biologic effect within the spread-out Bragg peak area is depth dependent.^55^*Abbreviation:* RBE = relative biologic effect.

LET is a crucial concept for the quantification of radiobiological effects. It has been observed that several parameters of ions, including RBE and oxygen enhancement ratio (OER), are primarily dependent on LET, among other factors. C-ions with therapeutic beams, usually within the range of 100 to 400 MeV/n, exhibit a LET that varies between 10 to 80 keV/µm, except for significantly higher values observed in the distal edge. The definition of RBE involves the calculation of the dose ratio of a test radiation that produces a similar biologic endpoint, typically cell death, in comparison to a reference radiation, which is commonly 250 kVp x-rays or Co-60 gamma rays.^13^ The RBE of C-ions exhibits variability and a positive correlation with depth, peaking at the distal edge of the Bragg peak. The generally accepted RBE for carbon ions used in clinical radiation therapy is typically estimated to range from 2.5 to 3. However, there have been reports of values as high as 5.^14^ The RBE of photons is consistently 1, regardless of their energy level. In contrast, the RBE of protons has been reported to be approximately 1.1, indicating a 10% increase in effectiveness compared with photons. The RBE holds promise in the field of radiation therapy, particularly for tumors that exhibit radioresistance due to a low α/β ratio. It is evident that a variable physical dose along the SOBP is necessary to maintain a constant biologic dose in the tumor due to the changes in RBE along the SOBP, as depicted in Figure 2. The biologic dose is determined by multiplying the absorbed dose, measured in Gray (Gy), with the RBE and is denoted as Gy (RBE).^15^

Particles with high ionization density result in intricate DNA damage. The phenomenon of complex DNA damage is frequently observed along the densely ionizing track of high-LET particles. This type of damage is typically considered to be resistant to repair due to the presence of multiple types of DNA lesions clustered nearby. The proximity of these lesions poses a challenge for a single DNA repair pathway to effectively resolve the damage. Because of the complex nature of DNA damage, its repair can be a challenging task. Consequently, heavy ion therapy is considered a potentially efficient approach to destroy tumors that are resistant to radiation and chemotherapy.^16^ It has been observed that therapeutic particles are effective in inducing cell death regardless of the cell cycle phase. This is attributed to the dense ionization pathway that occurs along the DNA.

The impact of oxygen partial pressure and LET on the OER is intricate. The objective is to evaluate the dosage required to achieve a comparable biologic outcome in the presence or absence of oxygen. OER estimates for carbon and other heavy ions show variability with respect to LET and may range from 2.5 to 1.0 depending on ion charge and LET.^17^ The efficacy of OER is significantly influenced by the dosage administered. Consequently, particles with high LET at suitable depths exhibit greater efficacy in eliminating cells in the hypoxic and necrotic cores of tumors in comparison to photons. This provides particle therapy with an additional biologic edge over photons. Heavy ions primarily exhibit direct effects, which reduces their reliance on the generation of free radicals and the availability of oxygen.

### Mechanism of killing cells in radiation therapy with carbon ions

The utilization of carbon ion radiation therapy might be a promising therapeutic alternative for individuals diagnosed with glioblastoma. This is attributed to its superior physical dose conformity and heightened biologic effectiveness in comparison to photons. Several studies have explored the mechanistic factors underlying the increased cellular mortality observed with carbon ions. The findings from in vitro investigations conducted on LN229 and U87 glioblastoma cell lines indicate that Carbon ions could elicit higher levels of the DNA double-strand break marker ƔH2AX compared with photons. Moreover, it was observed that the induction of this marker was particularly high for carbon ion doses that were <0.5 Gy. The increased RBE of carbon ion irradiation is mostly associated with complex DNA lesions. Cells exposed to double strands with repaired carbon ions break more slowly. Carbon ion irradiation has been shown to induce a stronger and longer G2 cell cycle arrest as well as a higher rate of apoptosis. The potential role of autophagy as a resistance mechanism in U87 cells after exposure to photon or carbon ion irradiation is negligible. The utilization of carbon ion radiation therapy may prove to be a valuable strategy in overcoming resistance mechanisms in phosphatase and tensin homolog-deficient glioblastoma. This approach involves the inhibition of nonhomologous end joining and the exploitation of homologous recombination suppression.^18^ The process of cell apoptosis plays a crucial role in the elimination of tumor cells by ionizing radiation. This is achieved through 2 primary signaling pathways, namely the extrinsic death pathway that involves the binding of death receptors and the intrinsic death pathway that is activated at the mitochondrion. Intrinsic apoptosis is believed to be a pivotal factor in modulating the vulnerability of neoplastic cells to radiation therapy through the activation of Caspase 1 or Caspase 3 genes.^19^^,^^20^ The impact of Poly(ADP-ribose) polymerase 1 on the radiation sensitivity of tumor cells has been documented in the literature. This is achieved through the modulation of DNA repair mechanisms, cell cycle, and autophagy after exposure to ionizing radiation.^21^^,^^22^ Caspase-independent cell apoptosis was found to be a significant compensatory mechanism in glioma cell death involving the polymerase 1/AIF (apoptosis-inducing factor) signaling pathway 24 hours after carbon ion exposure and was most likely caused by oxidative damage to DNA.^22^ Apoptosis signaling pathway in cervical cancer HeLa cells mediated by carbon ion irradiation has also been reported.^23^

Recent findings suggest that the resistance of tumors to radiation is influenced by the tumor microenvironment, which modulates the levels of various cytokines and growth factors, such as epidermal growth factor, vascular endothelial growth factor, basic fibroblast growth factor, and hepatocyte growth factor. Additionally, the microenvironment facilitates extracellular matrix degradation through matrix metalloproteinases.^24^^,^^25^ A study conducted by Liu et al aimed to assess the impact of carbon ion and x-ray radiation, as well as the tumor microenvironment, on the migratory behavior of glioma and endothelial cells. The authors proposed that the inhibition of cell migration induced by carbon ion radiation may occur through the activation of FAK signaling by vascular endothelial growth factor.^25^ Prior research has indicated that photons may have a role in the lack of treatment success by promoting the migration of tumor cells related to EGFR. The study conducted by Stahler et al revealed that carbon ion RT resulted in a decrease in the motility of glioblastoma multiforme cell lines. Additionally, the phosphorylation status of EGFR, AKT, and ERK1/2 did not exhibit any significant alterations upon exposure to carbon ion RT. This finding provides evidence for the advantageous impact of heavy ion irradiation in comparison to photon radiotherapy.^26^ Rieken and colleagues conducted a study to examine the impact of carbon ion irradiation on the migration of glioma cells U87 and Ln229. The study demonstrated that photon RT increases the likelihood of tumor cell migration and consequent promotion of locoregional spread through the induction of integrin expression by photons. Compared with photon radiation therapy, carbon ion radiation therapy has been observed to result in reduced integrin expression and inhibition of glioma cell migration on vitronectin and fibronectin substrates. This suggests that carbon ion radiation therapy may offer enhanced local control.^27^ The findings suggest that carbon ion radiation therapy may have a significant impact on the migration of glioma cells by reducing the expression of integrins on the cell surface.

### Latest clinical outcomes of treating glioblastoma tumors with carbon ions

In comparison to low LET radiation, such as photons, carbon ions exhibit a decreased fractionation effect in the normal tissues located within the clinical target volume (CTV). The diminished fractionation capability poses a significant obstacle in managing infiltrative neoplasms, including glioblastomas, which are among the prevalent primary brain tumors in the adult population. Glioblastomas are known to be radio-resistant and tend to progress rapidly. As a result, there is a possibility that carbon ions may have a significant role in the treatment of patients with glioblastoma. This section provides a comprehensive evaluation of the clinical trials that examined the efficacy of carbon ion therapy, both as a standalone treatment and in conjunction with photon or proton therapy, in patients with glioblastoma.

### Treatment of glioblastoma with carbon ions

Studies comparing CIRT to alternative treatment modalities, such as photon therapy, are detailed in this section. The findings indicated that severe cytotoxic effects were not observed as overall survival (OS), progression-free survival (PFS), and tumor local control improved. The utilization of carbon ion radiation therapy has been observed to exhibit a more pronounced dose-response relationship and a greater lethality toward glioblastoma cells.^28^ Carbon ions induce more pronounced and prolonged cell cycle delays, particularly in the G2 phase, which subsequently leads to the onset of mitotic catastrophe, induction of cellular senescence, and an elevated incidence of apoptosis and autophagy.^29^, ^30^, ^31^ In addition, the application of carbon irradiation has been observed to lead to a reduction in Integrin expression and consequent inhibition of glioma cell migration, thereby improving local control of the tumor.^27^ A study conducted by Lautenschlaeger et al aimed to examine the median survival rates of patients with recurrent glioblastoma in 2 different groups. The first group consisted of 40 patients who were reirradiated with photon (39 Gy in 13 fractions), whereas the second group consisted of 38 patients who were reirradiated with carbon ion (45 Gy RBE in 15 fractions). The log-rank test revealed a significant increase in median survival among patients who received carbon ion treatment compared with those who received photon treatment (8.0 vs 6.5 months, respectively). This difference may be attributed to the enhanced biologic effectiveness of carbon ions in hypoxic and necrotic tumors.^32^ The correlation between the dosage of carbon ions and its impact exhibits an escalation. In the phase 1/2 clinical trial, 14 patients with diffuse astrocytoma were treated with carbon ion radiation therapy administered in 24 fractions over 6 weeks. The patients were divided into 2 groups: a high-dose group consisting of 5 patients who received 55.2 GyE, and a low-dose group consisting of 2 patients who received 46.2 GyE and 7 patients who received 50.4 GyE. The results of the trial indicated that patients in the high-dose group exhibited a significant improvement in both PFS and OS rates compared with those in the low-dose group. Notably, no acute or late grade 3 or higher toxicities were observed in either group.^33^ Furthermore, the delivery and administration of carbon ion is safe and tolerable. In 118 patients, including 11 gliomas (glioblastoma n = 3), the initial toxicity of heavy ion particles 6 weeks after radiation therapy was evaluated at the Heidelberg Ion Therapy Center. Fifty-two patients received particle therapy alone, 48 patients received carbon ion, and 4 patients received proton ion. An additional 66 patients received particle therapy in the form of a carbon ion enhancement alongside advanced photon radiation therapy. No significant acute toxicity levels were discovered. All patients received the prescribed radiation therapy without interruption due to adverse effects. Mucositis, dysphagia, and erythema of the skin occurred most frequently in patients with head and neck cancer who received particle treatment as a boost and photon intensity modulated radiation therapy.^34^ Hasegawa et al also conducted an examination focusing just on visual acuity after exposure to carbon ions. No visual loss was observed in 35 individuals who received 57 GyE of irradiation to their optic nerves as part of an experiment on 54 patients with head and neck cancers treated with carbon ion irradiation and a median follow-up of more than 4 years. Contrarily, only 11 of the remaining 19 patients who were exposed to radiation doses more than 57 GyE experienced diminished visual acuity.^35^ The data presented indicate that the clinical application of carbon ions is safe for delivery. The notable enhancements suggest the commencement of a novel epoch in the field of radiation oncology.

### Treatment of glioblastoma with carbon ions and photon combination

The use of carbon ions in conjunction with photons to treat glioblastoma has been the subject of numerous studies. Even though some results indicate that the combination of CIRT and x-ray radiation therapy improves clinical outcomes, additional clinical studies are currently being conducted without published results. The RBE of carbon ions is higher than that of photons, with a range of 2 to 5, depending on the endpoint being studied and the glioblastoma cell line.^36^ The radiobiological effects of carbon ions in conjunction with low LET ionizing radiations have been the subject of numerous clinical studies due to the efficacy of carbon ions. In the first dose-escalation trial on 48 patients with malignant gliomas, x-ray irradiation was delivered to a total dosage of 50.0 Gy within 25 fractions, followed by carbon ion irradiation, according to a study carried out by the National Institute of Radiologic Sciences. Carbon ion dosages were raised in 8 portions over 2 weeks, from 16.8 to 24.8 GyE. The results showed that delivering greater cumulative doses of carbon ion boost after the first 50 Gy photon irradiation in 25 fractions results in a significant improvement in total survival duration.^37^ The novel approach of administering the CIRT boost before starting low-LET-based chemoradiotherapy is highly favored due to its ability to effectively target glioma stem cells, overcome hypoxic conditions, and modify the immunogenicity within the tumor microenvironment.^38^^,^^39^

In the context of multimodal treatment of primary glioblastoma, the Cleopatra trial is the first randomized trial to evaluate the effect of carbon ion radiation. Carbon ion (up to a total dose of 18 Gy E in 6 fractions) is compared with proton boost (up to a total dose of 10 Gy E in 5 individual fractions) when applied to a macroscopic tumor in the Phase II-Cleopatra-Study, which is performed after surgery at initial diagnosis in patients with glioblastoma who were treated with 48 to 52 Gy photon radiation. Temozolomide is administered to both arms. It is important to keep in mind that the outcomes of this clinical investigation are not yet finalized. The progression-free survival rate, overall toxicity, and overall patient safety are among the secondary objectives in addition to the primary endpoint, which is the overall survival rate at 12 months.^36^ Recurrent or progressive WHO grade II, III, or IV gliomas are being studied in the phase 1/2 Cinderella trial, comparing carbon ion radiation therapy with fractionated stereotactic radiation therapy. Patients with recurrent gliomas will be treated with 7 escalating dose regimens from 30 GyE in 10 fractions to 48 GyE in 16 fractions during phase 1 of the trial, at the end of which the recommended dose of carbon ion radiation will be determined. During phase 2, carbon ion or fractionated stereotactic radiation therapy (36 Gy in 18 fractions) is administered to patients in a nonselective manner. The principal objective of phase 1 is to assess toxicity, and the randomized part 2 aims to evaluate survival after reirradiation at the 12-month mark as its primary endpoint.^40^ The report of 7 patients who received a diagnosis of high-grade glioma, including primary and recurrent malignant astrocytoma and glioblastoma, was comprehensively outlined by the University of Heidelberg. These patients were among a cohort of 80 individuals with cancer who underwent treatment with heavy particles. Individuals diagnosed with WHO grade III primary astrocytoma and primary glioblastoma were administered treatment consisting of a carbon ion boost of 18 Gy E in 6 fractions, in addition to photon irradiation up to 50 Gy to the CTV. Patients with recurrent glioblastoma were treated with a total carbon ions dose of 36 Gy E administered in 12 fractions. Low-grade gliomas, on the other hand, were treated using protons in a standard 1.8 Gy fractionation. It is noteworthy to mention that all 80 patients were successfully treated.^41^ In 2012, the University of Heidelberg provided an update on their data regarding the treatment of gliomas in 26 patients through carbon ion irradiation. The total doses administered ranged from 18 to 45 GyE. After the process of randomization, protons having a range of total doses from 10 to 57.2 Gy E was chosen for the treatment of various cases, including low-grade meningioma, glioma, and one patient with glioblastoma, all of which involved children. The study investigated the efficacy of carbon ion or proton boost irradiation in combination with photon irradiation of 50 Gy for primary high-grade gliomas or high-grade meningioma. Additionally, the study examined the effects of particle treatment coupled with temozolomide-based chemotherapy in 17 individuals with gliomas. The administration of carbon ion radiation therapy, either as a treatment for recurrent glioblastoma or as a boost irradiation after prior photon therapy, resulted in a significant reduction in both tumor size and contrast agent uptake. In addition, after undergoing particle therapy, 50% of the 18 patients who received a diagnosis of glioblastoma experienced disease progression, ultimately leading to mortality in 44.4% of the cases.^42^ Combs et al conducted a study that highlights the efficacy of particle therapy and its potential to reduce the occurrence of secondary malignancies. The study in question involved the treatment of 176 patients with protons and 84 patients with carbon ions. Among the latter group, 36 patients received photon radiation therapy in addition to a carbon ion boost. In accordance with the CLEOPATRA protocol, WHO grade III gliomas and glioblastomas are treated concurrently with temozolomide (TMZ), 50 GyE photons, and 18 GyE carbon ion increase upon initial diagnosis. Mild acute side effects, such as alopecia, fatigue, headache, conjunctivitis, and skin erythema, were observed without unexpected severe toxicity (common toxicity criteria grade III). Unlike photon therapy, particle therapy appears to reduce immediate side effects such as hair loss and fatigue.^43^ In a phase 1/2 clinical trial involving 32 patients with glioblastoma multiform, Mizoe et al examined the positive effect of combining x-ray radiation therapy, chemotherapy, and carbon ion radiotherapy.^44^ Radiation therapy with conventional x-rays (2 Gy, 5 days per week) was combined with chemotherapy and followed by radiation therapy with carbon ions in the aforementioned study. Nimustine hydrochloride, at a dose of 100 mg/m^2^, was given in either week 1, week 4, or week 5 of x-ray radiation therapy. Patients who received a diagnosis of glioblastoma had a median survival time of 17 months. Patients treated with higher doses of carbon ion benefited from increased survival rates, suggesting that this combination therapy holds promise as a treatment for malignant glioma. Combination radiation therapy with temozolomide has been shown to be effective in preclinical studies, and its effects are cumulative and not time-dependent.^45^^,^^46^ Nonetheless, Combs et al conducted a clinical investigation of this, comparing patients who received a carbon ion boost in phase 1/2 trial (as in the aforementioned study by Mizoe et al) to patients who received either photons or photons combined with TMZ. Each treatment group consisted of 32 patients who received a diagnosis of glioblastoma and 16 individuals diagnosed with anaplastic astrocytoma. The study found that the median OS of patients who received a glioblastoma diagnosis was 9 months when treated with RT (photon therapy), 14 months when treated with radiochemotherapy (photon + chemotherapy), and 18 months when treated with (photon + C12). The overall survival of patients with glioblastoma was found to be improved by the addition of chemotherapy or carbon ions boost, compared with those who received only photon treatment. Therefore, they predicted the possible synergistic impact of coadministering C12 and TMZ. Furthermore, the synergistic effects of various chemotherapeutic agents, including paclitaxel and camptothecin, have been extensively demonstrated in numerous in vitro studies, with the most significant synergistic effects being observed.^38^

### Treatment of glioblastoma with carbon ions and proton combination

We discussed the benefits of using carbon ion and proton radiation in this section, including overall survival improvement and safety, especially for the treatment of radiation-resistant and hypoxic tumors, but due to the rarity of these studies, more clinical studies in this field are needed to reach a definite conclusion about the benefits of these radiations.

Proton beams have been found to offer more advantageous dose distributions in comparison to photon beams. This is due to the steep dose fall-off at the field borders, which enables more precise localization of dosage. Although protons possess a larger LET compared with photons, their radiobiological properties do not exhibit significant differences from those of photons.^47^ Although administering proton treatment at the same dose as photons may not enhance tumor control, it is possible that the risk of long-term toxicity could be reduced due to the lower integral dose.^48^ Because of the reduced exposure to the entire brain, proton beam therapy mitigates the risk of developing neoplasms induced by radiation therapy.^7^ Photon beam radiation consistently results in a higher equivalent uniform dose and secondary cancer risk.^49^ When a number of clinical trials are taken into consideration, carbon ions have demonstrated prospective advantages over protons due to their reduced lateral scattering, lower oxygen enhancement ratio, and higher relative biologic effectiveness. These factors make carbon ions a suitable candidate for the elimination of radio-resistant, hypoxic tumors,^50^ particularly when radiosensitive normal tissue surrounds the tumor.^47^ Carbon ions have demonstrated these prospective advantages over protons. The main dominance may relate to the possible reduction in long-term morbidity in cases of low-grade cerebral gliomas, but in cases of high-grade neoplasms, the use of modalities with more RBE may improve tumor control and patient survival.^48^ A randomized phase 1/3 trial conducted by The Shanghai Proton and Heavy Ion Center has investigated the collective impact of these massive particles. During the initial phase, the subjects were subjected to a carbon ion boost (3-6 GyE × 3) and were subsequently administered 75 mg/m^2^ temozolomide concurrently with 60 GyE of proton irradiation. Overall survival and toxicity rates were the major endpoints of the phase 3 trial, which randomly assigned patients with glioblastoma exclusively to receive either a carbon ion boost followed by 60 GyE proton with TMZ (experimental arm) or 60 GyE proton with TMZ without a carbon ion boost (control arm). They hypothesized that a 33% rise in OS rates might be achieved by inducing a carbon ion boost, which would increase the tumor-killing (including glioma stem cells) capacity. Furthermore, the rationale for adopting carbon as a boost in this study includes low toxicities in normal and nontargeted tissues due to rapid dosage fall off of carbon ion, delivering high LET carbon ion to CTV, and decreasing the influence of hypoxia in cell death.^38^ In 2020, the Shanghai Proton and Heavy Ion Center updated their findings from a phase 3 trial involving 369 patients with a fresh diagnosis of glioblastoma. Patients were randomly assigned to 1 of 3 categories:

First, (1) a control group received 60 Gy of photon radiation therapy, (2) Group A received proton radiation therapy (60 Gy), and (3) Group B received proton radiation (60 Gy) and a surge of carbon ions (15 GyE/3F). OS was set as the primary outcome, with PFS, side effects, and quality of life serving as secondary endpoints. Final outcomes from these studies will be made public by September 2025 (clinicaltrials.gov: https://clinicaltrials.gov/ct2/show/NCT04536649). According to reports, a significant hormonal dysfunction has been observed in patients with glioma who underwent photon radiation, even when the treatment field did not include the hypothalamus and pituitary. This correlation between photon and proton/carbon side effects has been studied in relation to gliomas. Particle therapy has the potential to reduce the risk of long-term complications, such as visual disturbances, neurocognitive deficits, and secondary malignancies.^48^ The comparative analysis of dose distribution among 4 conventional radiation techniques for treating head and neck tumors revealed that while photon intensity modulated radiation therapy (IMRT) resulted in a significantly higher dose to nontarget structures in glioblastoma patients, the dose distribution achieved through carbon ion therapy with raster scanning and proton therapy with active and passive scanning was found to be satisfactory.^51^ Numerous studies have demonstrated the viability and safety of proton and carbon ion therapies when used simultaneously.^10^ The first study to report survival outcomes after particle therapy and concurrent temozolomide was a study conducted by the Shanghai Proton and Heavy Ion Center on 34 glioblastoma multiform patients and 16 anaplastic glioma patients between June 2015 and October 2018 in which 24 patients received proton radiation therapy (at a dosage of 60 Gy E in 30 daily fractions), and 26 patients who had gross tumor after surgery/biopsy received proton radiation therapy in combination with carbon temozolomide was administered to all patients. The 12- and 18-month OS rates for patients with glioblastoma were 77.4% and 61%, respectively, and the comparable progression-free survival rates were 61.3% and 42.7%. During a median follow-up of 14.3 months, 29 patients developed grade 1 dermatitis/alopecia, and 7 exhibited pseudoprogression. Furthermore, 11 patients experienced grade 1 (n = 6) or grade 2 (n = 5) late severe effects of radiation-induced brain necrosis, with no grade 3, 4, or 5 acute or late toxicities. The safety and efficacy of particle irradiation at a dose of 60 GyE in patients with high-grade glioma were investigated.^52^ These findings are congruent with those of the Qiu et al investigation. When 4 doses of carbon ion boost (9, 12, 15, and 18 Gy RBE) in 3 fractions before proton radiation therapy (60 Gy RBE in 30 fractions) were administered to 16 glioblastoma patients and 2 anaplastic astrocytoma patients in a phase 1 trial, no severe (grade 3) acute or late toxicities were observed after a median follow-up of 17.9 months. The trial was halted because the first patient treated with 18 Gy RBE carbon ion boost developed grade 3 radiation necrosis. Patients with glioblastoma had 50.6% progression-free survival and 78.6% overall survival at 12 months. This study focused on the safety and efficacy of proton and carbon particles in patients with advanced gliomas.^39^ Despite the benefits of carbon and proton therapy, one of the most significant disadvantages of particle therapy is the high cost of its technical implementation as well as operation. Large cyclotrons or synchrotrons are necessary to accelerate protons and heavier ions to the required energy levels for the treatment of deep-seated malignancies.^47^

## Conclusion

The utilization of carbon ion radiation therapy, as opposed to photon radiation therapy, presents physical and radiobiological benefits that hold potential as a viable substitute for the management of gliomas. Recent clinical trials have demonstrated that carbon therapy, either as a standalone treatment or in conjunction with other modalities, has led to improved dose distribution at the tumor site, better local tumor control, and fewer complications, ultimately resulting in enhanced survival rates. Further research is required to explore the potential enduring adverse outcomes of this therapeutic approach.

## Disclosures

The authors declare that they have no known competing financial interests or personal relationships that could have appeared to influence the work reported in this paper.
